# Tibial component rotation in total knee arthroplasty

**DOI:** 10.1186/s12891-016-0940-z

**Published:** 2016-02-16

**Authors:** Peter Z. Feczko, Bart G. Pijls, Michael J. van Steijn, Lodewijk W. van Rhijn, Jacobus J. Arts, Peter J. Emans

**Affiliations:** Department of Orthopaedic Surgery, Maastricht University Medical Centre, P.O. Box 5800, 6202 AZ Maastricht, The Netherlands

**Keywords:** Total knee arthroplasty, Tibial rotation, ROM technique, TTL technique, Computer navigation

## Abstract

**Background:**

Both the range of motion (ROM) technique and the tibial tubercle landmark (TTL) technique are frequently used to align the tibial component into proper rotational position during total knee arthroplasty (TKA).

The aim of the study was to assess the intra-operative differences in tibial rotation position during computer-navigated primary TKA using either the TTL or ROM techniques. The ROM technique was hypothesized to be a repeatable method and to produce different tibial rotation positions compared to the TTL technique.

**Methods:**

A prospective, observational study was performed to evaluate the antero-posterior axis of the cut proximal tibia using both the ROM and the TTL technique during primary TKA without postoperative clinical assessment. Computer navigation was used to measure this difference in 20 consecutive knees of 20 patients who underwent a posterior stabilized total knee arthroplasty with a fixed-bearing polyethylene insert and a patella resurfacing.

**Results:**

The ROM technique is a repeatable method with an interclass correlation coefficient (ICC2) of 0.84 (*p* < 0.001). The trial tibial baseplate was on average 4.56 degrees externally rotated compared to the tubercle landmark. This difference was statistically significant (*p* = 0.028). The amount of maximum intra-operative flexion and the pre-operative mechanical axis were positively correlated with the magnitude of difference between the two methods.

**Conclusions:**

It is important for the orthopaedic surgeon to realise that there is a significant difference between the TTL technique and ROM technique when positioning the tibial component in a rotational position. This difference is correlated with high maximum flexion and mechanical axis deviations.

## Background

Rotational alignment of the components in total knee arthroplasty (TKA) is an important factor for both survival and the performance of the prostheses [1, 2]. The majority of the attention has focussed on the rotational alignment of the femoral component [3–6], which has resulted in the widespread use of the transepicondylar axis and the antero-posterior axis (Whiteside’s Line) of the distal femur as the reference axes for the rotational alignment of the femoral component [3–6].

However there is more discussion about the rotational alignment of the tibial component in part because of the difficulty of clinically assessing tibial component rotation. Furthermore, a whole range of anatomical landmarks can be used, including the medial border of the tibial tuberosity, the medial third of the tibial tuberosity, the anterior tibial crest, the posterior tibial condylar line, the second ray and the first web space of the foot. Aligning the tibial component to the tibial tubercle is one of the most popular landmark methods [7–9]. The disadvantage of all anatomical landmark techniques is that they do not account for femoro-tibial kinematics [10]. To address this problem, the ROM technique was introduced; in this technique, the rotational alignment of the tibial tray is determined through conformity to the femoral component when the knee is put through a series of full flexion-extension cycles [11]. However, the position of the tibial tray is not exclusively determined by the femoral component but is also influenced by the extensor mechanism, the patellar component, the ligament balancing and the tibial cut [12, 13]. The rationale behind the ROM technique lies in the theoretical advantage of aligning the tibial component in relation to the femoral component while respecting the soft tissue torsion forces to create optimal femoro-tibial kinematics [14]. For this method to work, the femoral component should be positioned accurately. Using computer-assisted surgery may improve the accuracy of positioning [15].

Several studies have demonstrated variability in the relationships between different landmarks and techniques for establishing rotational alignment of tibial components in total knee arthroplasty [11, 16–18]. A review reported that there is no gold standard measurement of tibial component rotation [18]. Whether the ROM technique is a repeatable method, and whether there is a significant difference in tibial component rotational position between the TTL technique and the ROM technique in computer navigated TKA with patella resurfacing remains unanswered questions in the literature. The primary purpose of this study was therefore to intra-operatively evaluate the repeatability of the ROM technique. The secondary outcome was to evaluate the difference in rotational alignment of the trial tibial component with the use of the TTL and ROM techniques during computer-navigated TKA with patella resurfacing . Additionally, the factors that influenced the positioning of the trial tibial component with both techniques were investigated. Postoperative clinical and radiological data were not collected.

## Methods

A prospective, observational study of 20 consecutive primary posterior stabilized TKAs in 20 patients with fixed-bearing polyethylene inserts (Scorpio Flex PS, Stryker Corporation, Mahwah, NJ USA) was performed by a single surgeon (MvS).

Data collection began with 10 consecutive TKAs to determine whether there was a difference between the alignment techniques and if so to gather data to perform a power analysis. Using the acquired data, we determined that a total of 20 subjects were needed to achieve 90 % power assuming a minimum detectable difference of 5.0 degrees, a standard deviation of 7.7 degrees and a significance level of alpha < 0.05.

The mean pre-operative mechanical leg axis was 3.65° ± SD 7.15 of varus. The mean pre-operative mechanical leg axis of the varus knees (*N* = 15) was 7.0° ± SD 4.05, of the valgus knees (*N* = 5) was −6.4° ± SD −4.15.

Positive values indicated varus alignment, negative values indicated valgus alignment.

The mean posterior slope was 1.5° ± SD 1.02.

### Ethics and consent

The Medical Ethics Committee of the Maastricht University Medical Centre has concluded that the described research does not apply to the Dutch Medical Research involving Human Subjects Act (WMO), therefore the patient was not required to provide consent regarding the use of the material.

Furthermore, every patient in the Maastricht University Medical Centre is provided with information regarding these kinds of studies. If they do not wish to contribute to these studies, this information will be included in their file. The patient involved in this study did not make an objection against the use of his/her material for research purposes.

### Patient demographics

The patient demographics are summarized in Table 1.

**Table 1:** Patient demographics. Positive values indicated varus alignment, negative values indicated valgus alignment

	Age (years)	Preoperative Leg axis (degrees varus)	Sex (Male/Female)	Side (Right/Left)	Preoperative ROM (degrees)	Preoperative flexion contracture (degrees)
Mean ± SD	69.8 ± 10	3.7 ± 7.2	8/12	10/10	117 ± 16	5.6 ± 4.7

### Operative procedure

The Stryker Knee Navigation System (Stryker Navigation System II, version 3.1) was used in this study. The prosthesis (Scorpio PS Stryker Howmedica Osteonics, Allendale, NJ USA) used in these surgeries allows five degrees of rotation between the tibial insert and the femoral component. In all cases, a tourniquet was applied for the entire duration of the surgery. After a standard midline skin incision and a medial parapatellar arthrotomy, the active wireless trackers of the navigation system were fixed to the femur and tibia. The required landmarks were entered into the navigation computer, and the rotation centre of the hip was determined by a special algorithm executed in customized software. The transepicondylar axis of the distal femur and the Whiteside’s line were set in each case exactly perpendicular to each other to improve the accuracy of the positioning the femoral component. The femoral component was aligned parallel to the transepicondylar axis. The AP axis of the proximal tibia was determined by placing the tip of the pointer on the centre of the line between the intercondylar eminences and aligning it to the medial 1/3 of the tibial tubercle . This AP axis was saved in the navigation program as 0 degrees of rotation. The proximal tibial and distal femoral cuts were performed and examined with the navigation system. The tibial posterior slope was set according to the patient’s natural slope. The polyethylene insert (Scorpio-Flex PS fixed bearing tibial insert) had an additional four degrees of posterior down-slope. The rotation of the femoral component was oriented according to the transepicondylar line and the AP axis (Whiteside’s Line) of the distal femur as currently advised in literature [3, 5, 17]. After soft tissue balancing and achievement of the maximal range of motion, the patella was prepared. The patellar button position may affect femoro-tibial kinematics; therefore, all of the trial components, including the patella and the PS tibial trial insert, were placed before the tibial component was subjected to the ROM technique. One navigation tracker was applied to the alignment handle of the trial tibial tray to check the position according to the given 0 degrees of rotation. Flexion and extension were measured intra-operatively after the approach was made and the trackers were placed. Positive values for extension represent hyperextension, while negative values represent flexion contracture.

The tibial component was inserted and checked for smooth movement on the tibial cut surface. The knee was then put through five full flexion-extension cycles while the surgeon held the ankle only. During the ROM cycles, no hands were touching the knee to prevent manual manipulation, and no varus/valgus stress was applied. The movement was followed on the navigation computer to confirm that no varus/valgus stress was applied. After performing the ROM cycles, the rotational position of the trial tibial component was recorded as indicated by the navigation computer. Positive values indicated that the trial component was in internal rotation according to the given TTL axis, and negative values indicated that the trial component was in external rotation. While this measurement was being acquired, the patella was lying in the patellar groove to facilitate optimal patellofemoral tracking and to prevent lateral pull on the patella tendon that could cause the tibia to rotate externally. The rotational position was noted (measurement 1). After removing and reinserting the components, the ROM technique was applied two additional times with five full flexion-extension movements and corresponding subsequent measurements from the navigation system (measurement 2 and 3).

After completing the operative procedure, the final tibial tray was cemented up to 1/3 of the medial border of the tibial tubercle.

### Statistics

The statistical analyses were performed with SPSS statistical software (Version 12, SPSS Inc., Chicago, IL USA). The reproducibility of the ROM-technique was evaluated using the intra-class correlation coefficient (ICC). For each target, there was one ‘rater’(MvS) who performed the three consecutive attempts at positioning the tibial component using the ROM technique. Since the exact same rater made ratings on every patient and it was assumed that both patients and observer were drawn randomly from larger populations, the ICC2 was used [19]. The ICC2 reflects the reliability of this single rater. Means were compared with paired T-tests in cases of normal distributions [20]. A level of *p* < 0.05 was considered statistically significant. The mean of the 3 ROM measurements was used to evaluate the difference between the ROM and TTL techniques. We evaluated potential factors associated with the difference between the ROM and TTL technique including, leg axis, intra-operative flexion, intra-operative extension and posterior slope. (Table 2) The associations between each variable and the difference between the ROM and TTL were examined with univariable regression analyses. Factors that were associated with the outcome in univariable analyses (*p*-values < 0.20) were included in multivariable regression analyses. In multivariable regression analyses *p*-values < 0.05 were considered significant. Regression coefficients with their 95 % confidence intervals are reported.

**Table 2:** Outcome and available covariates assessed for inclusion in the regression model. Positive values for extension represent hyperextension, while negative values indicate flexion contracture

Variables	Mean ± SD	(range)
Difference ROM and TTL (°)	−4.6 ± 8.6	(−27.0 to 11.5)
Varus mechanical leg axis (°)	3.7 ± 7.2	(−13.0 to 15.0)
Intra-operative flexion (°)	121.9 ± 6.7	(108.0 to 134.0)
Intra-operative extension (°)	−0.1 ± 3.0	(−5.5 to 7.0)
Posterior slope (°)	1.5 ± 1.0	(0.0 to 3.5)

## Results

The tibial component can be reliable positioned in terms of rotation using the ROM technique, as demonstrated by an ICC2 = 0.84 (95 % CI (0.70–0.93); *p* < 0.001). The ICC2 = 0.84 of tibial component positioning using the ROM technique indicating nearly perfect repeatability. Because the ROM technique was nearly perfectly reliable, the means of the 3 ROM measurements were used to evaluate the difference between the two techniques. With the ROM technique, the tibial component was on average 4.56 (± SD 8.59) degrees externally rotated compared to the tubercle landmark. This difference was statistically significant *p* = 0.028.

It appeared from the multivariable regression analyses that more valgus pre-operative mechanical leg axis (−0.54 (95%CI −0.98 - -0.10); *p* = 0.019), intra-operative flexion (0.57 (95%CI 0.13 – 1.00); *p* = 0.014) and intra-operative extension (1.41 (95%CI 0.50 – 2.32); *p* = 0.005) were associated with a greater difference between tibial component positioning using the ROM and TTL techniques. (Table 3) These results indicate that increasing the pre-operative varus mechanical leg alignment by 1 degree resulted in an increase in the external rotation of the tibial component of 0.54 degrees relative to the tibial tubercle using the ROM technique.

**Table 3:** Results of univariable and multivariable regression analyses

Variable	Univariable analysis		Multivariable analysis	
	coefficient (95%CI)	*p*-value	coefficient (95%CI)	*p*-value
Varus mechanical leg axis	−0.58 (−1.09 to −0.05)	0.030	−0.54 (−0.98 to −0.10)	0.019
Intra-operative flexion	0.82 (0.33 to 1.31)	0.002	0.57 (0.13 to 1.00)	0.014
Intra-operative extension	1.19 (−0.09 to 2.48)	0.070	1.41 (0.50 to 2.32)	0.005
Posterior slope	2.63 (−1.31 to 6.56)	0.180	0.04 (−3.05 to 3.13)	0.979

In the varus knees, the tibial component was on average 5.9 (± SD 8.7) degrees externally rotated; in the valgus knees, the mean external rotation was 0.4 (± SD 7.6) degrees relative to the tibial tubercle. The differences between the rotational alignments using the ROM and TTL techniques in both the varus and valgus knees were not statistically significant (*p* = 0.221).

The cut posterior slope (0.04 (95%CI −3.05 – 3.13); *p* = 0.979) was not significantly related to the difference in the rotational alignment of the tibial component according to the tibial tubercle in either the ROM or TTL techniques.

## Discussion

This study revealed that the ROM technique is a repeatable method for aligning the tibial component. Using the ROM technique, the tibial component was on average 4.56 (± SD 8.59) degrees externally rotated compared to the tubercle landmark. Our result is in contradiction with Ikeuchi et al. [11] They found that using the ROM technique results in a more internally rotated position of the tibial component en found also widely variable results. However in line with our findings, Berhouet [21] and Chotanaputhi [22] found the ROM technique reproducible and the alignment of the tibial tray was externally rotated in comparison with the medial border of the tibial tubercle and the posterior tibial condyle line respectively. Ikeuchi [11] used cruciate-retaining (CR) TKA components without patellar resurfacing. The mean posterior slope of the cut tibia was 5 degrees and they compared the position of the tibial component related to the Akagi line [23]. The amount of tibial slope, the design of the prosthesis and the patella resurfacing all might have influenced the outcome. Rossi [24] stated that the ROM technique is a reproducible method to establish tibial rotation during TKA, having found that components were positioned in 0.35 external rotation to the Akagi line.

Using anatomical landmarks for the rotational alignment of the femoral and tibial components is a widely accepted method. Alignment to the medial 1/3 of the tibial tubercle (Insall’s reference [25]) is based on papers of Nicoll [26], Lawrie [27], Lützner [28] and Yin [29], who found the medial 1/3 of the tibial tubercle the most accurate and reliable anatomical landmark. However, determining the component positions separately can lead to rotational mismatch between the femoral and tibial components [30]. Using the dynamic ROM technique may allow for the tibial tray to align itself according to the femoral component position, ligament balancing and extensor mechanism alignment.

Retrieval and biomechanical studies have indicated that femoro-tibial rotational mismatches cause increased contact stress on the tibial insert and patellar component that leads to accelerated polyethylene wear [31–33]. Steinbrück et al. [34] recommended the rotational alignment of the tibial component to the medial 1/3 of the tibial tubercle to achieve the lowest retro-patellar pressure. Using the ROM technique the tibial component was externally rotated by a mean 4.56 degrees respect the tibial tubercle which might have resulted in increased retro-patellar peak pressure. Kim et al [35] found the best survival rate when tibial component was aligned between 2 degrees of internal rotation to 5 degrees of external rotation to the medial 1/3 of the tibial tubercle. External rotational errors were not associated with pain in a study of Nicoll [26].

To measure the rotational difference between the ROM and TTL techniques in degrees may not be easy during TKAs that are performed without navigation. Computer navigation is an ideal method for measuring the difference in trial tibial tray position between the TTL and ROM techniques given its reported accuracy of one degree [36]. Furthermore, the use of computer navigation can help to optimize the femoral component position, which is crucial for the performance of the ROM technique. [15, 37] Computer navigation has no advantage regarding the identification of the correct positions of the anatomical landmarks, but it does have advantages in comparing the positions of the landmarks to other landmarks (e.g., the transepicondylar axis to Whiteside’s line) and positioning the implants according the identified landmarks.

Huddleston et al. [16] found that when the ROM technique is applied to varus knees, the antero-posterior axis of the tibial tray is significantly more externally rotated then when this technique is applied to valgus knees. The same result was found in our study, although this difference did not reach statistical significance likely because our study was underpowered regarding this aspect. However, the pre-operative mechanical leg axis was correlated with the difference between the ROM and TTL techniques (*p* = 0.019). With increasing pre-operative varus alignment, the ROM technique results in increasing external rotation of the tibial component.

The maximum degrees of flexion (*p* = 0.014) and extension (*p* = 0.005) during surgery were also correlated with the difference between the ROM technique and the tubercle landmark in this study, which indicates that the use of the ROM technique for a patient with great preoperative flexion would result in a more internally rotated tibial component position compared with a patient with less preoperative flexion.

### Limitations of the study

The number of patients (observations) in our study is rather low. Various studies have suggested that for each variable studied in multiple regression analysis at least 10 observations are required [38–40] although a recent study showed that this number could be lower in certain circumstances [41]. The results should therefore be interpreted with some caution.

Clinical and radiological data were not collected postoperatively. Therefore, no results are available regarding potential differences in clinical outcomes between the two techniques. It is known that a tourniquet affects the intra-operative patello-femoral tracking [42, 43]. Therefore, it is likely that the tourniquet had some effect on the tibial component rotational alignment in the ROM technique. The use of a tourniquet during all of the operations and keeping it inflated while performing the ROM cycles might have affected our results.

Although tibial rotational alignment is also effected by ligament balancing, we did not measure the gaps intra-operatively.

The design of the prosthesis (CR or PS version) and the design of the tibial tray (symmetric or anatomical) may also have influence on the outcome [44].

## Conclusions

The ROM technique is a repeatable intra-operative method for determining the rotational position of the tibia trial component. Because the best method to determine the intra-operative position of the tibia component is still under debate, TKA surgeons should be aware that there is a difference between the ROM and TTL methods, particularly in patients with high peri-operative ranges of motion and/or high pre-operative varus/valgus alignment.
